# Prevalence, Molecular Characterization, and Antimicrobial Resistance Profiles of Shiga Toxin-Producing *Escherichia coli* Isolated from Raw Beef, Pork, and Chicken Meat in Vietnam

**DOI:** 10.3390/foods13132059

**Published:** 2024-06-28

**Authors:** Hoang Minh Duc, Cam Thi Thu Ha, Tran Thi Khanh Hoa, Le Van Hung, Nguyen Van Thang, Hoang Minh Son

**Affiliations:** 1 Department of Veterinary Public Health, Faculty of Veterinary Medicine, Vietnam National University of Agriculture Trau Quy, Gia Lam, Hanoi 12400, Vietnam; 2 Veterinary Hospital, Faculty of Veterinary Medicine, Vietnam National University of Agriculture Trau Quy, Gia Lam, Hanoi 12400, Vietnam; 3 Department of Anatomy and Histology, Faculty of Veterinary Medicine, Vietnam National University of Agriculture, Trau Quy, Gia Lam, Hanoi 12400, Vietnam

**Keywords:** Shiga toxin-producing *Escherichia coli*, ESBL, colistin, meat

## Abstract

Shiga toxin-producing *Escherichia coli* (STEC) is one of the most important foodborne pathogens, and the rise of antibiotic resistance to it is a significant threat to global public health. The purpose of this study is to investigate the prevalence, molecular characterization, and antibiotic resistance of STEC isolated from raw meat in Vietnam. The findings in this study showed that the prevalence of STEC in raw beef, pork, and chicken meat was 9.72% (7/72), 5.56% (4/72), and 1.39% (1/72), respectively. The STEC isolates were highly resistant to ampicillin (91.67%) and tetracycline (91.67%), followed by trimethoprim/sulfamethoxazole (83.33%), streptomycin (75%), and florfenicol (66.67%). The incidence of STEC virulence-associated genes, including *stx_1_*, *stx_2_*, *eae*, and *ehxA*, was 8.33% (1/12), 91.67% (11/12), 33.33% (4/12), and 58.33% (7/12), respectively. STEC serogroups O157, O26, and O111 were detected in 3 out of 12 STEC isolates. Two isolates were found to be ESBL producers carrying the *bla*_CTX-M-55_ gene, and three isolates were colistin-resistant strains harboring the *mcr-1* gene. Notably, a STEC O111 isolate from chicken meat harbored both the *bla*_CTX-M-55_ and *mcr-1* genes.

## 1. Introduction

Shiga toxin-producing *Escherichia coli* (STEC) is recognized as one of the most dangerous foodborne pathogens that can cause severe symptoms, including diarrhea, bloody diarrhea, hemorrhagic colitis (HC), and life-threatening conditions such as haemolytic uraemic syndrome (HUS) and thrombotic thrombocytopaenic purpura (TTP) [1,2]. The severity of STEC infection is known to be serotype-dependent. Although more than 500 STEC serotypes have been identified, a few serogroups, such as O157 and the non-O157 groups O26, O45, O103, O111, O121, and O145, have been previously reported to be associated with HC, HUS, and TTP [3]. Yearly, STEC infections are responsible for more than 265,000 illnesses in the United States [4,5] and 2.8 million acute infections with 4000 cases of HUS worldwide [6]. It has been estimated that 36% of all STEC infections in humans were linked to O157 [5,7]. Meanwhile, the “Big Six” STEC serogroups (O26, O45, O103, O111, O121, and O145) accounted for 71% of STEC infections [8]. The treatment cost per STEC infection case ranged from USD 26 to USD 211,084 depending on the severity of infection [9].

STEC, part of the normal microflora in cattle intestines, can contaminate beef during improper slaughter, making beef a potential transmission vector [10,11,12]. Pork and chicken meat have also been reported as contaminated with STEC due to environmental factors and mishandling during processing [12,13,14,15]. In addition, previous studies have reported that STEC contamination has led to significant financial losses due to the recall of meat products [16].

Antimicrobial resistance (AMR) poses a global threat to human and animal health [17,18], with the World Health Organization (WHO) recently reporting at least 700,000 deaths annually due to AMR [19]. This number could rise to 10 million deaths per year by 2050 [17,19]. The overuse and misuse of antibiotics in both clinical and veterinary settings have been attributed to the emergence of antibiotic resistance, leading to the loss of antibiotic efficacy and the limitation of antibiotic options for treating bacterial infection [20,21]. When treating a STEC infection, the administration of antibiotics must be carefully considered, as certain antibiotics such as ciprofloxacin and trimethoprim/sulfamethoxazole can enhance the production of the Shiga toxin (Stx). Therefore, only a few antibiotics such as azithromycin, fosfomycin, and meropenem are recommended for the treatment of early-stage STEC infections, as they have been shown to effectively eliminate the pathogen, prevent the release of the Shiga toxin, and reduce the risk of kidney failure. As a result, treating antibiotic-resistant STEC infections poses significant challenges. Furthermore, STEC can transfer mobile plasmid-containing antibiotic-resistant genes to commensal *E. coli*, thereby transforming susceptible strains into antibiotic-resistant ones [22].

Despite the growing concern regarding the antibiotic resistance of STEC, there is a lack of information on the antibiotic resistance profile of STEC isolated from meat. This study aims to determine the prevalence, molecular characteristics, and antibiotic resistance profile of STEC isolated from raw beef, pork, and chicken meat sold at wet markets and supermarkets in Vietnam.

## 2. Materials and Methods

### 2.1. Sample Collection

A total of 216 meat samples (72 beef, 72 pork, and 72 chicken) were randomly collected from wet markets (108 samples) and supermarkets (108 samples) in Hanoi from July 2022 to May 2023. Samples were transported to the laboratory in sterile bags and ice boxes within 24 h.

### 2.2. Prevalence of STEC in Beef, Pork, and Chicken Meat

Each 25 g meat sample was homogenized using a Seward stomacher 400 circulator (Seward Ltd., Worthing, UK) in 225 mL of modified EC broth with novobiocin and incubated at 42 °C for 18-24 h. After enrichment, DNA was extracted from 1 mL of the culture using the DNeasy tissue kit (Qiagen, Hilden, Germany) following the manufacturer’s instructions. The DNA was screened for *stx_1_* and *stx_2_* genes using PCR with the primers listed in Table 1 [23].

The PCR was performed in a 25 µL reaction volume, comprising 2.5 μL of 10× PCR Buffer, 10 µL of 1 mM dNTPs, 5 µL of 5U Taq polymerase, 1 µL of each primer (5 μM), 2 μL of DNA template, and 1.5 µL of deionized water.

The PCR thermal cycling included an initial denaturation at 95 °C for 5 min, followed by 35 cycles at 94 °C for 30 s, 59.4 °C for 80 s, and 68 °C for 75 s, and a final elongation at 68 °C for 7 min. Post-amplification, the PCR product was analyzed by 2% agarose electrophoresis and viewed under ultraviolet light using a BioRad Molecular Imager^®^ GelDocTM XR (BioRad Laboratories, Hercules, CA, USA).

*Stx*-positive enriched cultures were used for the isolation of STEC. Briefly, 5 mL of the culture was inoculated into 45 mL of MacConkey broth (Oxoid Ltd., Hants, UK) and then incubated at 37 °C for 13 h. After incubation, a portion of the sample (1 mL) was withdrawn and serially diluted with Phosphate-Buffered Saline (PBS; 137 mM NaCl, 8.10 mM Na_2_HPO_4_, 2.68 mM KCl, 1.47 mM KH_2_PO_4_) before being spread on CHROMagar O157 (CHROMagar, Paris, France) supplemented with cefixime (0.05 mg/L) and tellurite (2.5 mg/L). The plates were then incubated overnight at 37 °C. The following day, up to 30 colonies exhibiting the typical morphology of *E. coli* were randomly selected for colony PCR to detect *stx* genes using the same primers as mentioned above. *Stx*-positive colonies were then re-streaked on CHROMagar O157. Afterward, presumptive STEC colonies on CHROMagar O157 were confirmed again via PCR. Finally, STEC isolates were preserved at −86 °C for further characterization.

### 2.3. Detection of STEC Virulence-Associated Genes and Serogroup-Specific Genes

STEC isolates were subjected to multiplex PCR for the detection of accessory virulence genes (*eae* and *ehxA*), and serogroups-specific genes (*rfb_O157_*, *wzx_O**145**_, wzqE_O**121**_/wzqF_O**121**_, wzx_O**111**_, wzx_O**103**_, wzx_O**45**_*, and *wzx_O**26**_*) following the same method as mentioned above, with some modifications [23]. The primers used in this assay are presented in Table 1.

Each PCR reaction was conducted in a volume of 25 μL, consisting of 2.5 μL of 10× PCR Buffer, 10 µL of 1 mM dNTPs, 5 µL of 5U Taq polymerase, 0.5 μL of each O111 primer (10 μM), 0.25 µL each of other primers (10 µM; O157, O145, O121, O103, O45, O26, eae, and ehxA), 2 μL of DNA template, and 0.5 µL of deionized water. The PCR amplification program used in this experiment was the same as that used for the detection of *stx* genes.

### 2.4. Antimicrobial Susceptibility Profile of STEC Isolates

STEC isolates were selected for the antimicrobial susceptibility test using broth dilution methods according to the guidelines of the Clinical and Laboratory Standards Institute (CLSI) [24]. Fifteen antibiotics were used in this study, including ampicillin, tetracycline, streptomycin, gentamicin, colistin, azithromycin, trimethoprim/sulfamethoxazole, florfenicol, meropenem, cefotaxime, cefoxitin, cefepime, ceftazidime, ciprofloxacin, and nalidixic acid. *E. coli* strain ATCC 25922 served as a quality control strain. Isolates showing resistance to at least 1 antibiotic from 3 or more antibiotic classes were classified as multidrug-resistant strains.

Isolated STEC strains resistant to cefotaxime and/or ceftazidime were specifically chosen to assess their ability to produce extended-spectrum *β*-lactamase (ESBL), using an ESBL test as previously described by CLSI [24].

### 2.5. Detection of ESBL and Colistin-Resistant Genes

STEC isolates capable of producing ESBL were tested for the presence of *β*-lactamase-encoding genes (ESBL genes) using multiplex PCR according to the previously described method [25]. The primers for detecting ESBL genes are shown in Table 2.

The volume of each PCR reaction was 25 µL, consisting of 2.5 μL of 10× PCR Buffer, 5 µL of 1 mM dNTPs, 2.5 µL of 5U Taq polymerase, 0.5 uL of each primer (50 μM), 2 μL of DNA template, and 7 µL of deionized water.

The PCR amplification conditions were as follows: an initial denaturation at 95 °C/5 min, followed by 25 cycles of denaturation at 95 °C for 30 s, annealing at 60 °C for 90 s, extension at 72 °C for 90 s, and a final extension at 68 °C for 10 min. PCR amplicons were then visualized according to the procedure mentioned above. For further molecular characterization, PCR products were amplified and sequenced with the Sanger method using an Applied Biosystems 3500 genetic analyzer (ABI 3500, Applied Biosystems, Waltham, MA, USA). The nucleotide sequences were then analyzed using the BLASTP [26] and Resfinder-3.1 server [27].

Colistin-resistant STEC isolates were also subjected to multiplex PCR for detecting *mcr* genes, including *mcr-1, mcr-2, mcr-3, mcr-4,* and *mcr-5* [28]. Primers used for the screening of *mcr* genes are displayed in Table 3. The PCR reaction mixture (25 μL) contained 12.5 µL of DreamTaq Green PCR Master Mix (Thermo Fisher Scientific, Waltham, MA, USA), 5.5 µL of deionized water, 0.5 µL each of all primers (10 µM), and 2 µL of DNA template. The thermal cycler condition used for this assay comprised an initial denaturation at 94 °C for 5 min, followed by 25 cycles of denaturation at 94 °C for 30 s, annealing at 58 °C for 90 s and 72 °C for 1 min, and a final elongation at 72 °C for 10 min. After amplification, the PCR product was analyzed as described above.

## 3. Results

### 3.1. Prevalence of STEC in Beef, Pork, and Chicken Meat

The PCR results in Table 4 showed that *stx* genes were detected in all meat types tested at the rate of 16.2% (35/216). The highest positive rate of *stx* was found in beef (23.61%; 17/72), followed by pork (15.28%; 11/72), and then chicken meat (9.72%; 7/72). The *stx_2_* gene (12.96%; 28/216) was more frequently detected than *stx_1_* (4.17%; 9/216). Only two samples (0.93%; 2/216) tested positive for both the *stx_1_* and *stx_2_* genes.

The findings in Table 4 also revealed that 12 (5.56%) out of 216 meat samples tested were positive for STEC. The prevalence of STEC in beef, pork, and chicken meat was 9.72% (7/72), 5.56% (4/72), and 1.39% (1/72), respectively. A total of 21 STEC strains were isolated from 12 *stx*-positive samples. Among 21 STEC isolates, 2 (9.5%) carried *stx_1_* and 19 (90.5%) harbored *stx_2_*. None of the STEC isolates carried both the *stx_1_* and *stx_2_* genes. A STEC strain harboring *stx_1_* was isolated from the chicken meat sample, while STEC strains harboring *stx_2_* were isolated from the beef and pork samples. Overall, the prevalence of STEC in meat purchased in wet markets (6.48%; 7/108) was found to be higher than in supermarkets (4.63%; 5/108).

### 3.2. Detection of STEC Virulence-Associated Genes and Serogroup-Specific Genes

To avoid duplicates, only one STEC isolate per meat sample was selected to detect STEC virulence-associated genes and serogroup-specific genes and to determine antimicrobial resistance. Table 5 displays the virulence-associated gene profile of the 12 STEC isolates selected. The *eae* gene was detected in four (33.33%; 4/12) STEC isolates, including two from beef, one from pork, and one from chicken meat samples. Meanwhile, *ehxA* was found in seven (58.33%; 7/12) STEC isolates. Among them, five STEC strains were recovered from beef, one from pork, and one from chicken meat samples. Three out of twelve STEC isolates were serotypeable, belonging to three different serogroups, including O157, O26, and O111. On the contrary, the remaining nine isolates were unserotypeable. The STEC O157 strain was recovered from beef samples and carried *stx_2_*, *eae*, and *ehxA* genes. STEC O26, co-harboring *stx_2_* and *eae,* was isolated from pork samples. STEC O111 isolate of chicken meat origin simultaneously carried *stx_1_*, *eae*, and *ehxA*.

### 3.3. Antimicrobial Susceptibility Profile of STEC Isolates

The antibiotic resistance profile of 12 STEC isolates is detailed in Table 5 and Table 6. The STEC strains isolated in this study exhibited the highest resistance rates to ampicillin (91.67%) and tetracycline (91.67%), followed by trimethoprim/sulfamethoxazole (83.33%), streptomycin (75%), and florfenicol (66.67%). In contrast, the lowest resistance rates were observed with respect to cefotaxime, ceftazidime, cefepime, gentamicin, and ciprofloxacin, all at a rate of 16.67% (2/12). None of the STEC isolates were found to be resistant to cefoxitin and meropenem. The results also showed that all STEC isolates were resistant to at least two antibiotics. In particular, the STEC O111 strain isolated from chicken meat in this study resisted 13/15 antibiotics tested. Ten (83.33%) out of twelve STEC isolates were identified as multidrug-resistant strains. In addition, two isolates were identified as ESBL producers, three were colistin-resistant, and one was resistant to both colistin and cephalosporins.

### 3.4. Detection of ESBL and Colistin-Resistant Genes

The results of multiplex PCR showed that two phenotypically confirmed ESBL isolates carried *bla*_CTX-M1_ group genes; a subsequent sequencing assay confirmed that these genes were *bla*_CTX-M-55_. Additionally, all three colistin-resistant STEC isolates harbored *mcr-1* genes. In particular, the STEC O111 isolate carried both *bla*_CTX-M-55_ and *mcr-1* genes.

## 4. Discussion

This study is the first report on the prevalence of STEC in raw beef, pork, and chicken meat in Vietnam. Our results showed that, respectively, 9.72%, 5.56%, and 1.39% of raw beef, pork, and chicken meat samples were contaminated with STEC. The findings in this study were higher than our previous study conducted in Japan, which reported that the prevalence of STEC in raw beef, pork, and chicken meat was 7.2%, 3.8%, and 0%, respectively [12]. A lower prevalence of STEC in meat was also observed in a study performed in Switzerland, where 5/211 (2.3%) minced beef samples and 2/189 (1%) minced pork samples tested positive for STEC [30]. In a study carried out in the USA, STEC was detected in 2 (5.2%) of 231 ground pork and 13 (5.2%) of 249 ground beef samples [31]. A higher prevalence of STEC was noted in a study in Iran, reporting that 29.70% of beef samples were contaminated with STEC [32]. Similarly, an investigation by Barlow et al. on the prevalence of STEC in retail meat in Australia showed that STEC was found in 46/285 (16%) ground beef and 111/275 (40%) lamb samples [33]. Egervan et al. examined the occurrence of STEC in the Swedish retail market. The results indicated that 6 (2.0%) of the 300 samples of Swedish beef, 17 (13%) of the 135 samples of beef from other EU countries, and 6 (14%) of the 42 samples of beef from countries outside of the EU were contaminated with STEC [34]. The differences in the prevalence of STEC may be due to the different collection methods, sample sizes and seasons, isolation protocols, and geographic locations [15].

Serogroups are important factors in determining the pathogenicity of STEC. STEC O157 has been widely recognized as the most common STEC serogroup responsible for human illness globally [35]. Among non-O157 STEC serogroups, O26 has been identified as the predominant one associated with human disease worldwide [36]. Meanwhile, STEC O111 has been reported as the primary cause of HUS [8]. In this study, only three of the twelve isolates were serotypeable, with O157, O26, and O111 being the identified serogroups. O157 was detected in raw beef samples, while O26 and O111 were found in pork and chicken meat samples, respectively. These findings align with the data from the Europe Food Safety Authority (EFSA) and the European Centre for Disease Prevention (ECDC), showing that the STEC serogroups most frequently detected in beef in Europe were O157 and O26, followed by O148, O145, O8, O113, O91, O130, O174, and O113 [37]. A study conducted in South Africa investigating the prevalence of STEC serogroups in swine feces indicated that 31.81% (7/22) of STEC isolates belonged to serogroup O26, while the remaining isolates were unserotypeable [38]. Although serogroup O26 was frequently reported in pork product chains in the U.S., Europe, and Africa [15], it has not been documented in Asia. As for STEC O111, this serogroup has been previously isolated in beef [11,39] and pork [40] but not in chicken meat. Overall, the current study is the first report on the occurrence of STEC O26 in pork and STEC O111 in chicken meat in Vietnam.

Virulence-associated genes are another crucial parameter for identifying the pathogenicity of STEC. Among various virulence factors, the Shiga toxin (Stx) has been known to play the most important role in damaging endothelial cells and causing HUS [41]. Stx has two types: Stx1, encoded by the *stx_1_* gene, and Stx2, encoded by the *stx_2_* gene. STEC strains may harbor *stx_1_*, *stx_2_*, or both. It has been observed that STEC strains carrying *stx_2_* were more frequently linked to HC or HUS as compared to those harboring *stx_1_* alone or both *stx_1_* and *stx_2_* [12,42]. In our study, 11 out of 12 STEC isolates carried only the *stx_2_* gene, indicating their potential to cause severe diseases. This finding is consistent with previous research that has reported a high prevalence of STEC strains harboring only the *stx2* gene in meat [12,43]. While the Shiga toxin is a key virulence factor, it is not solely responsible for causing diseases. Additional virulence factors, such as intimin and enterohemolysin, are also necessary for STEC infection [44,45]. Intimin, encoded by *eae,* facilitates the attachment of STEC strains to the host’s intestine, leading to lesions in intestinal epithelial cells. The presence of the *eae* gene has been reported to enhance the pathogenicity of STEC [46]. In our study, 33.33% (4/12) of STEC isolates tested positive for the *eae* gene, consistent with the previous findings in China (34.69%) and Japan (20%) [12,47]. Plasmid-carried enterohemolysin encoded by *ehxA* is a heat-labile, pore-forming toxin that can cause the hemolysis of host red blood cells. STEC strains harboring the *ehxA* gene have been known to be associated with HUS [48]. In the current study, we found that 58.33% (7/12) of STEC isolates possessed the *ehxA* gene, indicating their high pathogenicity.

In Vietnam, antibiotics such as ampicillin, tetracycline, trimethoprim/sulfamethoxazole, streptomycin, and florfenicol have been used extensively for many years in animal husbandry for animal disease prevention and treatment and growth promotion [49]. This could partly explain the high resistance rates (66.67%-91.67%) of STEC isolates to these antibiotics and the high MDR rate (83.33%; 10/12) observed in the present study. Similar results were recorded in a study conducted by Ahmed et al. in Egypt, where 31 out of 54 (57.4%) STEC O157:H7 isolates of food origin were MDR, with high resistance rates to kanamycin (96.8%), followed by spectinomycin (93.6%), ampicillin (90.3%), streptomycin (87.1%), and tetracycline (80.6%) [50]. Another study reported that STEC isolates of human, animal, and food origins resisted streptomycin, ampicillin, and tetracycline at high rates of 82.35%, 72,54%, and 43.13%, respectively [51]. In addition, Novella et al. found that 65% of STEC isolated from humans and cattle in Brazil were identified as MDR strains, exhibiting the highest resistance rates to tetracycline (100%), streptomycin (78.1%), and trimethoprim/sulfamethoxazole (56.2%) [52].

Both extended-spectrum cephalosporins and polymyxins (colistin) belong to the critically important antimicrobial group [53]. However, the effectiveness of these antibiotics is declining due to their excessive and inappropriate use in livestock [54]. A survey conducted by Carrique-Mas et al. (2014) and Dang et al. (2013) in Vietnam indicated that colistin was commonly used for disease prevention, disease treatment, and growth promotion [55,56]. Another investigation into antimicrobial usage in livestock in Vietnam also showed that beta-lactam (25%), polymyxin (20.7%), and macrolide (17.4%) were the most frequently used antibiotic classes in pig farms, while the most popular antibiotic classes in chicken farms were beta-lactam (27.6%), followed by tetracyclines (20.9%) and polymyxins (13.5%) [57]. In this study, we found that two STEC isolates were third-generation cephalosporins-resistant STEC isolates carrying the *bla*_CTX-M-55_ gene and that three isolates were colistin-resistant strains harboring the *mcr-1* gene. In particular, the STEC O111 isolate possessed both the *bla*_CTX-M-55_ and *mcr-1* genes. A study conducted by Bai et al. in China also reported that a STEC O116:H11 isolate of pig origin was resistant to 20 different antibiotics and carried both the *bla*_CTX-M-55_ and *mcr-1* genes [58]. This study represents the first documentation of the co-existence of the *bla*_CTX-M-55_ and *mcr-1* genes in a STEC O111 strain isolated from chicken meat in Vietnam. The appropriate use and management of antibiotics on farms is essential to reduce the transmission of antibiotic-resistant bacteria to meat.

## 5. Conclusions

In summary, the present study is the first to report on the prevalence, molecular characteristics, and antibiotic resistance profile of STEC isolates of meat origin in Vietnam. Overall, the incidence of STEC in meat in Vietnam was 5.56%, with most STEC isolates showing resistance to multiple antibiotic classes and testing positive for the *stx_2_* gene. Notably, one STEC isolate co-carrying the *bla*_CTX-M-55_ and *mcr-1* genes was also found in this study. Taken together, the data of our study suggest that meat could serve as a potential source of antibiotic-resistant STEC and emphasize the importance of developing intervention strategies.

## Figures and Tables

**Table 1:** Primer for detecting STEC virulence-associated genes and serogroups-specific genes.

Target Gene	Primer	Primer Sequence	Amplicon Size (bp)
*stx_1_*	stx1-F	TGTCGCATAGTGGAACCTCA	655
	stx1-R	TGCGCACTGAGAAGAAGAGA	
*stx_2_*	stx2-F	CCATGACAACGGACAGCAGTT	477
	stx2-R	TGTCGCCAGTTATCTGACATTC	
*rfb_O157_*	O157-F	CAGGTGAAGGTGGAATGGTTGTC	296
	O157-R	TTAGAATTGAGACCATCCAATAAG	
*wzx_O45_*	O45-F	GGGGCTGTCCAGACAGTTCAT	890
	O45-R	TGTACTGCACCCAATGCACCT	
*wzx_O103_*	O103-F	GCAGAAAATCAAGGTGATTACG	740
	O103-R	GGTTAAAGCCATGCTCAACG	
*wzqE_O121/_wzqF_O121_*	O121-F	TCAGCAGAGTGGAACTAATTTTGT	587
	O121-R	TGAGCACTAGATGAAAAGTATGGCT	
*wzx_O145_*	O145-F	TCAAGTGTTGGATTAAGAGGGATT	523
	O145-R	CACTCGCGGACACAGTACC	
*wzx_O26_*	O26-F	AGGGTGCGAATGCCATATT	417
	O26-R	GACATAATGACATACCACGAGCA	
*wzx_O111_*	O111-F	TGCATCTTCATTATCACACCA	230
	O111-R	ACCGCAAATGCGATAATAACA	
*eae*	eae-F	CATTATGGAACGGCAGAGGT	375
	eae-R	ACGGATATCGAAGCCATTTG	
*ehxA*	ehxA-F	GCGAGCTAAGCAGCTTGAAT	199
	ehxA-R	CTGGAGGCTGCACTAACTCC	

**Table 2:** Primers for detecting *β-*lactamase-encoding genes.

Target Gene	Primer	Primer Sequence	Amplicon Size (bp)
*bla*_TEM_	TEM-F	GGTCGCCGCATACACTATTCTC	372
	TEM-R	TTTTATCCGCCTCCATCCAGTC	
*bla*_SHV_	SHV-F	CCAGCAGGATCTGGTGGACTAC	231
	SHV-R	CCGGGAAGCGCCCTCCAT	
*bla*_CTX-M-1_	CTX-M1-F	GAATTAGAGCGGGAGTCGGG	588
	CTX-M1-R	CACAACCCAGGAAGCAGGC	
*bla*_CTX-M-2_	CTX-M2-F	GATGGCGACGCTACCCC	107
	CTX-M2-R	CAAGCCGACCTCCCGAAC	
*bla*_CTX-M-9_	CTX-M9-F	GTGCAACGGATGATGTTCGC	475
	CTX-M9-R	GAAACGTCTCATCGCCGATC	
*bla*_CTX-M-8/25_	CTX-M8/25-F	GCGACCCGCGCGATAC	186
	CTX-M8/25-R	TGCCGGTTTTATCCCCG	

**Table 3:** Primers for the detection of *mcr* genes.

Target Gene	Primer	Primer Sequence	Amplicon Size (bp)	Reference
*mcr-1*	*mcr1* f	AGTCCGTTTGTTCTTGTGGC	320	[28]
	*mcr1* r	AGATCCTTGGTCTCGGCTTG		
*mcr-2*	*mcr2* f	CAAGTGTGTTGGTCGCAGTT	715	[28]
	*mcr2* r	TCTAGCCCGACAAGCATACC		
*mcr-3*	*mcr* 3 f	AAATAAAAATTGTTCCGCTTATG	929	[28]
	*mcr3* r	AATGGAGATCCCCGTTTTT		
*mcr-4*	*mcr4* f	TCACTTTCATCACTGCGTTG	1116	[28]
	*mcr4* r	TTGGTCCATGACTACCAATG		
*mcr-5*	*mcr5* f	ATGCGGTTGTCTGCATTTATC	1644	[29]
	*mcr* 5 r	TCATTGTGGTTGTCCTTTTCTG		

**Table 4:** Prevalence of *stx* genes and STEC in meat.

Market Type	Meat Type	No. of Samples Tested	Detection of *stx* Genes in Meat Samples				Isolation of STEC			
			Number of Positive Samples (%)	No. of Samples Positive for Gene (%)			Number of Positive Samples (%)	No. of STEC Positive for Gene		
				*stx_1_*	*stx_2_*	*stx_1_* and *stx_2_*		*stx_1_*	*stx_2_*	*stx_1_* and *stx_2_*
Wet Market	Beef	36	9 (25)	2 (5.56)	8 (22.22)	1 (2.78)	4 (11.11)	0	7	0
	Pork	36	6 (16.67)	0 (0)	6 (16.67)	0 (0)	2 (5.56)	0	3	0
	Chicken	36	6 (16.67)	2 (5.56)	5 (13.89)	1 (2.78)	1 (2.78)	2	0	0
	Total	108	21 (19.44)	4 (3.7)	19 (17.59)	2(1.85)	7 (6.48)	2	10	0
Supermarket	Beef	36	8 (22.22)	3 (8.33)	5 (13.89)	0 (0)	3 (8.33)	0	6	0
	Pork	36	5 (13.89)	2 (5.56)	3 (8.33)	0 (0)	2 (5.56)	0	3	0
	Chicken	36	1 (2.78)	0 (0)	1 (2.78)	0 (0)	0 (0)	0	0	0
	Total	108	14 (12.96)	5 (4.63)	9 (8.33)	0 (0)	5 (4.63)	0	9	0
Total	Beef	72	17 (23.61)	5 (6.94)	13 (18.06)	1 (1.39)	7 (9.72)	0	13	0
	Pork	72	11 (15.28)	2 (2.78)	9 (12.5)	0 (0)	4 (5.56)	0	6	0
	Chicken	72	7 (9.72)	2 (2.78)	6 (8.33)	1 (1.39)	1 (1.39)	2	0	0
	Total	216	35 (16.2)	9 (4.17)	28 (12.96)	2 (0.93)	12 (5.56)	2	19	0

**Table 5:** Virulence-associated genes and antibiotic resistance patterns of STEC isolates.

Meat Type	Serotype	*stx* Gene	Accessory Virulence Gene		Antibiotic Resistance Pattern
			*eae*	*ehxA*	
Beef (*n* = 7)	UST	*stx_2_*	+	+	AMP-STR-TET-FLO-STX
	UST	*stx_2_*	−	−	AMP-TET
	UST	*stx_2_*	−	−	TET-FLO-STX
	UST	*stx_2_*	−	+	AMP-STR-TET-STX
	UST	*stx_2_*	−	+	AMP-STR-TET-NAL-STX
	O157	*stx_2_*	+	+	AMP-GEN-STR-TET-CST-FLO-AZM-STX
	UST	*stx_2_*	−	+	AMP-CTX-FEP-CAZ-STR-TET-FLO-CIP-NAL-STX
Pork (*n* = 4)	UST	*stx_2_*	−	+	AMP-AZM
	O26	*stx_2_*	+	−	AMP-STR-TET-FLO-STX
	UST	*stx_2_*	−	−	AMP-STR-TET-FLO-STX
	UST	*stx_2_*	−	−	AMP-STR-TET-CST-FLO-NAL-STX
Chicken (*n* = 1)	O111	*stx_1_*	+	+	AMP-CTX-FEP-CAZ-GEN-STR-TET-CST-FLO-AZM-CIP-NAL-STX

UST, unserotypeable; (+), Positive; (−), Negative; AMP, ampicillin; CTX, cefotaxime; FEP, cefepime; CAZ, ceftazidime; TET, tetracycline; STR, streptomycin; GEN, gentamicin; AZM, azithromycin; FLO, florfenicol; SXT, trimethoprim/sulfamethoxazole; CIP, ciprofloxacin; NAL, nalidixic acid; CST, colistin.

**Table 6:** Antibiotic resistance profile of STEC isolates.

Antibiotic Class	Antibiotic	No. of Isolates (Resistance Rate %)			
		Beef (*n* = 7)	Pork (*n* = 4)	Chicken (*n* = 1)	Total (*n* = 12)
Penicillins	ampicillin	6 (85.71)	4 (100)	1 (100)	11 (91.67)
	cefoxitin	0 (0)	0 (0)	0 (0)	0 (0)
Cephalosporins	cefotaxime	1 (14.29)	0 (0)	1 (100)	2 (16.67)
	ceftazidime	1 (14.29)	0 (0)	1 (100)	2 (16.67)
	cefepime	1 (14.29)	0 (0)	1 (100)	2 (16.67)
Cabarpenems	meropenem	0 (0)	0 (0)	0 (0)	0 (0)
Polymyxins	colistin	1 (14.29)	1 (25)	1 (100)	3 (25)
Aminoglycosides	gentamicin	1 (14.29)	0 (0)	1 (100)	2 (16.67)
	streptomycin	5 (71.43)	3 (75)	1 (100)	9 (75)
Tetracyclines	tetracycline	7 (100)	3 (75)	1 (100)	11 (91.67)
Phenicols	florfenicol	4 (57.14)	3 (75)	1 (100)	8 (66.67)
Macrolides	azithromycin	1 (14.29)	1 (25)	1 (100)	3 (25)
Fluoroquinolones	ciprofloxacin	1 (14.29)	0 (0)	1 (100)	2 (16.67)
Quinolones	nalidixic acid	2 (28.57)	1 (25)	1 (100)	4 (33.33)
Sulfonamides	trimethoprim/ sulfamethoxazole	6 (85.71)	3 (75)	1 (100)	10 (83.33)

## Data Availability

The original contributions presented in the study are included in the article, further inquiries can be directed to the corresponding author.
